# The role of gender inclusive leadership during the COVID-19 pandemic to support vulnerable populations in conflict settings

**DOI:** 10.1136/bmjgh-2020-003760

**Published:** 2020-09-21

**Authors:** Kristen Meagher, Neha S Singh, Preeti Patel

**Affiliations:** 1 Department of War Studies, King's College London – Strand Campus, London, UK; 2 Department of Global Health and Development, London School of Hygiene & Tropical Medicine, London, UK

**Keywords:** health policy, public health

**Summary box**

- Lessons learned from previous disease outbreaks in conflict settings should be harnessed to mitigate gendered impacts of COVID-19 on populations in conflict-affected countries.
- During a pandemic, resources for and access to adequate health services are often disrupted due to armed conflict.
- Pandemics are a gendered vulnerability, with their socioeconomic impact disproportionately higher among women particularly in conflict settings, where this vulnerability is exacerbated.
- Increased diversity and gender-balanced leadership is an essential requirement in key committees and in multilateral organisations in developing pandemic preparedness and responses.
- Intentionally cultivating and amplifying female leadership is paramount to creating effective leadership models and gender inclusive responses to improve outcomes for vulnerable populations in conflict settings.

## Background

‘*The real heroines in the fight against COVID-19 are women*’.^1^ Significant attention has been given to women political leaders in high-income settings, where it has been reported that women have led several countries’ effective national responses to COVID-19.^2^ However, little attention has been given to the role of women as leaders and decision makers in conflict settings. In conflict settings, COVID-19 is a multidimensional and existential crisis for many: a pandemic colliding with poor governance, insecurity, instability, other disease outbreaks (eg, cholera), disintegrated health and education systems, and food insecurity.^3^ These have dire consequences for vulnerable populations in conflict settings, including women and girls.^4^ Pandemics are a gendered vulnerability, with their socioeconomic impact disproportionately higher among women.^5 6^ In this article, we argue that cultivating and harnessing the advancements of women’s leadership globally and implementing a gender inclusive lens in pandemic preparedness and responses by including the experiences and voices of women in conflict settings is paramount. This will in turn create effective leadership models, as well as improving women and girls’ access to adequate healthcare in conflict settings.

## Women and girls are especially vulnerable to COVID-19 in conflict-affected settings

Women and girls are disproportionately affected by armed conflict and humanitarian emergencies.^7^ This disproportionality has been exacerbated during COVID-19, where in conflict settings one of the most affected and at-risk population groups include women and girls who lack decision-making power.^8^ Analysis from UN Women identifies five critical areas that leave women and girls most vulnerable during COVID-19, including: increased risks for sexual and gender-based violence (SGBV) in the context of pandemic response policies; unemployment; economic and livelihood impacts for the poorest women and girls; unequal distribution of care and domestic work; and women and girls’ voices not being included for an informed and effective response.^9^ Women’s and girls’ predominant role in caregiving, and as health and social welfare responders, also makes them particularly exposed to potential contamination.^10^ In conflict settings, conflict itself promotes conditions during which existing gender inequalities and inequities are amplified; community structures, access to healthcare and human rights are all compromised resulting in worsening conditions for women.^11^

During a pandemic, resources for and access to adequate health services can be further complicated by armed conflict.^12 13^ Of particular concern, resources to deal with the pandemic, as evidenced during Ebola and Zika, are often diverted from essential health services for women and girls, namely sexual and reproductive health, with lasting effects for themselves, their children, their families and their economies.^14^ Previous public health emergencies have shown that the impact of an epidemic on sexual and reproductive health often goes unrecognised, because the effects are often not the direct result of the infection but instead the indirect consequences of strained healthcare systems, disruptions in care and redirected resources.^15^ A study modelling three scenarios on the indirect effects of COVID-19 on maternal and child mortality in low-income and middle-income countries in which the coverage of essential maternal and child health interventions is reduced by 9.8%–51.9%, and the prevalence of wasting is increased by 10%–50% over 6 months would result in 253 500 additional child deaths and 12 200 additional maternal deaths.^16^ The most severe scenario—coverage reductions of 39.3%–51.9% and wasting increase of 50%—over 6 months would result in 1 157 000 additional child deaths and 56 700 additional maternal deaths.^17^ Furthermore, the pandemic is also impacting family planning, due to closure of health facilities or their inability to provide these services, disrupted supply chains and community outreach efforts (eg, via mobile clinics), and women and girls not being able to attend these clinics or facilities. The United Nations Population Fund (UNFPA) has predicted that 47 million women of the 450 million currently using modern contraceptives in low-income and middle-income countries will be unable to use them, with an additional 2 million women unable to use them for every additional 3 months that the lockdown continues.^18^ Altogether, these secondary impacts of the pandemic will be devastating to the autonomy and mental, physical and economic well-being of women, thus further undermining gender equality and equity.

SGBV also increases during humanitarian crises, and access to support services are frequently halted or disrupted.^19^ While it has been extensively reported that SGBV against women increases in non-conflict settings, it is challenging to obtain SGBV data in conflict settings during COVID-19, and it is widely under-reported.^20^ Research shows that an increase in SGBV was observed during the 2013–2015 Ebola outbreak in West Africa, as response efforts focused on containing the disease.^21^ The International Rescue Committee has found through an analysis of its gender-based violence (GBV) case management data that the suspension of these protection services for women, restrictions on mobility, lack of information and increased isolation and fear have resulted in a dramatic drop in the number of reported cases of violence against women and girls in conflict settings, including Syria, Iraq and Burkina Faso.^22 23^ Modelling from UNFPA predicts that COVID-19 is likely to cause a one-third reduction in progress towards ending GBV by 2030, including 31 million additional GBV cases expected as a result of 6-month lockdowns.^18^ Furthermore, the UNFPA analysis reports that a 2-year delay in initiating prevention programmes is projected to lead to an additional 13 million child marriages, as well as 2 million female genital mutilation cases over the next decade that otherwise would have been averted, that is, a 33% reduction in progress.

Testing capabilities for COVID-19 are also challenging in resource-scarce settings, many of which are affected by conflict. Of imminent concern, Yemen, Chad, Nigeria, Mali and Northern Syria especially have low testing numbers, highlighting the dangerous prospect of undetected and therefore uncontrolled COVID-19 outbreaks.^24^ Furthermore, much of the data being ascertained is missing critical information, that is, data disaggregated by sex or age. The global average denotes that 51% of cases are male. Yet, in places where armed conflict is occurring including Somalia, Pakistan, Chad, the Central African Republic, Afghanistan and Yemen, COVID-19 positive cases are more than 70% male.^25^ While testing is extremely limited across all these countries, this could point to an even greater lack of access to testing and healthcare for women in conflict-affected countries, despite increased exposure to the disease as primary caregivers and healthcare workers.^24^

Policies implemented in response to COVID-19 reveal significant gendered impacts that are exacerbated in conflict settings. Quarantine measures pose significant risks for women and children experiencing domestic abuse, and for those already in precarious settings, the risk of domestic and sexual abuse is exacerbated.^26^ Policies of social distancing, self-isolation, hygiene measures, including increased use of personal protection equipment, shielding and quarantining are all very resource intensive. What does this mean in communities where many live in close proximity in camp settings or similar? What does ‘self-isolation’ mean for those internally displaced by conflict? Even families and individuals who have money to buy food are finding it difficult to prepare for ‘lockdowns’, so what will be the fate of those who cannot afford to buy food because they are unable to go out to work? For the most vulnerable, lockdown measures make providing support to these individuals significantly more challenging.

## Leadership and decision making

Diverse, inclusive leadership is urgently required at local, national and global levels to improve pandemic preparedness and responses in conflict settings and mitigate their gendered impacts. Various recommendations have been suggested since the emergence of COVID-19 to create gender-inclusive responses, including engaging women frontline workers, women’s groups and networks in all decision making and policy spaces to improve health security surveillance, detection and prevention mechanisms.^9^ In 2019, the Global Preparedness Monitoring Board called for the involvement of more women in planning and decision making as a vital part of sustainable outbreak preparedness efforts.^27^ Yet analysis of recent emergencies clearly demonstrates little has been done to ensure that women’s voices are included in decision-making responses. Drawing lessons from previous disease outbreaks, namely the 2014–2016 West Africa Ebola and Zika, women were less likely than men to have power in decision making around the outbreak and their specific needs, resulting in their health needs being largely unmet.^28 29^

The multilateral system plays a critical role in establishing women’s rights and gender equality as a global norm; ‘anything that undermines the multilateral system has a negative impact on women and on their position in society’.^30^ Despite these lessons and recommendations, decision-making bodies established specifically for COVID-19 have not always reflected gender balance. In January 2020, only seven women were invited to join the 21-member WHO Emergency Committee on COVID-19.^31^ The WHO’s more recent decision to appoint Ellen Johnson Sirleaf and Helen Clark to lead the Independent Panel for Pandemic Preparedness and Response is, however, encouraging for the promotion of more women in leadership in health in conflict settings, given the experiences of these women.^32^

Women’s representation and engagement in leadership roles would put women and girls’ issues at the forefront of the global agenda, challenge the traditional hierarchies of knowledge and power by highlighting undervalued and unrecognised knowledge and advocate for more inclusive, diverse and representative decisions.^33 34^ Recognising women’s achievements, as both contributors and leaders, in the response to COVID-19 will aid in creating positive role models for others and is a pragmatic advocacy tool to advance the role of women as leaders and decision makers.^3^ While the theory that men and women have distinctly different leadership styles is an outdated concept, the idea that women perform better as leaders during crises has been purported in gender analysis of leadership and discussed widely during the COVID-19 pandemic.^2 35^ Experience shows that a systematic and intentional gender lens leads to more effective local, national and global responses and management of infectious disease outbreaks: women’s leadership and contributions are critical to curbing infection rates and enabling resilience and recovery.^9^ This is vital in conflict settings to reduce inequalities, which require ‘special attention’ through ‘hands on, exemplary leadership’.^1^ Diverse, inclusive leadership should therefore be seen as a central pillar of the global response to COVID-19, particularly in countries impacted by conflict. Leadership must go beyond a position or title; decision-making power is critical. Guidelines, frameworks and subsequent implementation and practice of these must be gender inclusive.

Building on this, women do not form a homogenous group; therefore, when women are excluded in decision making and policy implementation, other groups are also disadvantaged.^36^ Intersectional analysis places power at the centre and takes a broad approach to conceptualising how power hierarchies and systemic inequalities shape an individual’s life experience, thereby recognising that intersecting oppressions shape the experiences of individuals.^37^ As outlined earlier, it is clear that these intersecting oppressions are heightened in conflict settings. Therefore, feminist approaches to leadership should include an intersectional approach. While it is known that women from low-income and middle-income countries comprise just 5% of leadership positions in global health organisations,^38^ in conflict-affected countries, there is no substantive data available on the number of women in global health leadership positions. Therefore, by making national and international policy spaces truly representative, substantive participation of women and individuals from minority caste, religious, ethnic backgrounds could positively impact the health of millions in the future.^39^

## Conclusion

Advancements in gender equality across the globe risk being derailed by the COVID-19 pandemic; this will likely be further exacerbated in countries impacted by conflict as evidence suggests, women and girls living in conflict-affected countries are particularly vulnerable to both the direct and indirect impacts of COVID-19.^40^ Despite working overwhelming on the frontline as health workers, the diverse needs of women, and subsequently their families and communities, are often not met as they are not included in decision-making processes. Therefore, advocating for more women as leaders and decision makers at all levels in conflict settings is crucial to adequately address the gendered complexities of pandemics to better support vulnerable populations.
